# Bottom-Up Communication Approach for Effective Implementation of the One Health Initiative in Ethiopia

**DOI:** 10.12688/f1000research.166948.1

**Published:** 2025-09-03

**Authors:** Tadesse Shiferaw Chekol, Samuel Degie Abebe, Metasebia Tegegn Baheru

**Affiliations:** 1 One Health, Armauer Hansen Research Institute, Addis Ababa, Addis Ababa, 1005, Ethiopia; 2 Health Information and Counseling, Federal Ministry of Health, Addis Ababa, Addis Ababa, 1234, Ethiopia; 3 Health Science, Addis Ababa University College of Health Sciences, Addis Ababa, Addis Ababa, 9086, Ethiopia

**Keywords:** One Health, Bottom-up Communication, Ethiopia

## Abstract

The One Health approach, which integrates human, animal, environmental, and plant health, is Crucial for addressing complex public health challenges in Ethiopia. It addresses zoonotic diseases, antimicrobial resistance, and ecological degradation. These issues have increased the national burden alongside efforts for infectious disease prevention and control. However, effective implementation relies on communication strategies that are participatory, context-specific, and responsive to local realities. This paper presents a bottom-up communication framework designed to enhance local ownership, strengthen multisectoral collaboration, and align policy with community needs. The proposed approach emphasizes inclusive stakeholder engagement, the capacity-building of frontline actors, and the establishment of continuous feedback loops connecting community, district, and national levels to ensure the adaptive and sustainable implementation of One Health in Ethiopia.

## Introduction

One Health is an integrated and unifying approach that aims to sustainably balance and optimize the health of humans, animals, plants, and ecosystems. It recognizes that the health of humans, domestic and wild animals, plants, and the broader environment (including ecosystems) are closely linked and interdependent. ^1^ Ethiopia faces a complex array of interconnected health challenges that underscore the urgent need for a robust One Health approach. The country continues to struggle with endemic zoonotic diseases such as rabies, anthrax, and brucellosis, along with periodic outbreaks of emerging infectious diseases like Rift Valley fever and avian influenza. The growing burden of antimicrobial resistance (AMR), exacerbated by the unregulated use of antibiotics in both the human and animal health sectors, is another one health issue. The country has prioritized five zoonotic diseases: rabies, anthrax, brucellosis, leptospirosis, and echinococcosis. ^2^ Currently, Mpox is another newly emerging health threat that requires special attention. These issues are further exacerbated by rapid population growth, environmental degradation, and high levels of human-animal-environment interaction, particularly in pastoral and agropastoral communities, where livelihoods heavily depend on livestock and natural resources. In recognition of these threats, Ethiopia has made significant strides in developing national-level frameworks to institutionalize the One Health approach. This includes establishing the One Health Steering Committee, formulating the One Health Strategic Plan (2022–2026), and fostering cross-sectoral collaboration among the Ministries of Health, the Ministry of Agriculture, and the Environment Commission. ^3^ However, the practical implementation of these frameworks remains uneven and often limited in reach. One of the primary barriers is the dominance of top-down communication structures that prioritize centralized decision-making and technical directives while overlooking local knowledge, cultural dynamics, and the practical realities of frontline actors. Research and global experiences increasingly demonstrate that participatory, bottom-up communication strategies are critical to the success of One Health initiatives. These strategies help build trust between communities and institutions, encourage meaningful behavioral change, and facilitate coordination among sectors that have historically operated in silos. ^4, 5^ Community-based health surveillance that integrates human and animal health has shown promise in Ethiopia’s remote pastoral areas. Studies in the Somali Regional State demonstrated that engaging local leaders and community health workers in syndromic surveillance, supported by mobile technology, improves early detection and reporting of human and animal health events. The One Health Surveillance and Response System (OHSRS) approach, involving multiple sectors and community participation, enhanced zoonotic disease detection and response. ^6^ Community-based animal health workers (CAHWs) have been crucial in delivering veterinary services in remote areas, though sustainability remains a challenge. ^7^ The integration of priority zoonotic disease surveillance into existing polio eradication programs has shown success, with community volunteers reporting disease alerts and reaching numerous households. However, challenges such as delayed integrated monitoring mechanisms persist. ^8^ These initiatives demonstrate the potential of community-based approaches in strengthening local health surveillance and response systems. This paper, therefore, explores the rationale for a bottom-up communication approach and proposes a practical framework for its integration into Ethiopia’s One Health system.

## The rationale for bottom-up communication in one health

Communication is defined as the structured process of exchanging information to achieve shared understanding and coordinated action. It plays a pivotal role in enabling cross-sector collaboration and community engagement within One Health systems. It is acknowledged not only as an operational necessity but also as a strategic enabler of coordination, engagement, learning, and adaptive response in multisectoral One Health implementation. ^1^ Bottom-up communication strategies are increasingly seen as essential for the successful implementation of complex policy frameworks such as the One Health initiative, particularly in decentralized and multisectoral contexts like Ethiopia. This approach empowers local stakeholders by incorporating their lived experiences, knowledge systems, and cultural contexts into decision-making processes, thereby enhancing the relevance, acceptability, and sustainability of interventions. In rural Ethiopian communities, where zoonotic diseases frequently emerge, local actors are often the first to observe and respond to health threats; yet, they remain underrepresented in formal surveillance systems and policy dialogues. ^9^ Bottom-up communication cultivates trust in institutions and scientific initiatives, which is critical for effective risk communication and compliance during public health emergencies. ^10^ It also supports innovative and context-specific solutions by fostering continuous feedback, learning, and problem-solving among community members, health workers, and government actors. Furthermore, this approach facilitates both horizontal collaboration among sectors (such as health, agriculture, and environment) and vertical integration across administrative levels, from the community up to national policymaking bodies. Without inclusive communication mechanisms, implementation efforts risk becoming top-down directives that fail to resonate with or be adopted by the very populations they aim to serve. Therefore, bottom-up communication is not merely an engagement tool but a strategic necessity for realizing the full potential of the One Health approach in Ethiopia.

## Institutional considerations and policy alignment

Effective implementation of a bottom-up communication approach in Ethiopia’s One Health framework requires institutional coordination, decentralization, and alignment with existing service delivery systems. While the national One Health Steering Committee provides strategic direction, weak linkages between federal, regional, and community levels limit the flow of local insights into national planning. ^11^ Integrating One Health into Ethiopia’s decentralized governance system, particularly through health, agriculture, and environmental offices at the woreda level, is essential.

A practical entry point is the Health Extension Program (HEP), which has a wide community presence but operates largely in isolation from veterinary and environmental services. ^12^ Establishing interdisciplinary teams, bringing together health extension workers, animal health assistants, and environmental officers, can strengthen collaboration at the grassroots. Initiatives such as the Oromia rabies control program have shown that coordinated community-level responses improve both efficiency and trust.

Policy alignment is also critical. While Ethiopia’s One Health Strategic Plan (2022–2026) outlines multisectoral goals, operational gaps remain due to fragmented data systems, siloed budgets, and unclear mandates. ^13^ Strengthening joint planning and reporting mechanisms, particularly at regional and district levels, can enhance coordination. Universities and research institutions can play a key role here, as seen in projects like the One Health data integration pilot at Jimma University.

To institutionalize this approach, existing legal and regulatory frameworks should be updated to formally recognize intersectoral collaboration. Empowering regional bureaus with the authority and resources to coordinate locally appropriate One Health actions, especially in high-risk border areas, will improve responsiveness. Finally, creating incentives for community-level innovation and feedback, such as flexible budgets and participatory monitoring tools, can reinforce local ownership and sustain the bottom-up model.

## Challenges and mitigation strategies

Implementing a bottom-up communication approach within Ethiopia’s One Health framework faces several practical challenges. First, limited infrastructure, such as poor internet access, inadequate transportation, and weak mobile networks, hinders timely information flow between communities and institutions, particularly in remote pastoral areas. Second, human resource gaps exist at the community level, where health and veterinary workers often lack cross-sectoral training and coordination mechanisms. Additionally, vertical and horizontal fragmentation across ministries leads to parallel systems, duplicative efforts, and policy misalignment.

Another significant barrier is the lack of clear legal mandates for multisectoral coordination, which reduces accountability and limits the formal recognition of local actors’ contributions. Inconsistent data sharing and weak surveillance integration across human, animal, and environmental health systems also delay early detection and response efforts. Lastly, community engagement remains underdeveloped, with limited structures for incorporating grassroots perspectives into planning and feedback loops. ^14^

To mitigate these challenges, targeted investments should prioritize expanding digital infrastructure in underserved regions and strengthening the capacity of frontline workers through interdisciplinary training. Embedding One Health teams within existing structures, such as health posts and woreda offices, can improve integration and resource sharing. Updating national laws and operational guidelines to formalize cross-sectoral collaboration will provide institutional clarity and enable better coordination. At the community level, introducing low-cost, culturally appropriate communication tools (e.g., local radio, mobile alerts, and participatory forums) can enhance information flow and foster engagement. Ultimately, addressing these challenges will require a deliberate, coordinated effort to bridge system-level gaps while empowering local actors to lead context-specific solutions.

## Bottom-Up communication framework

The integrated bottom-up communication model for One Health in Ethiopia is designed to move beyond abstract coordination by embracing grounded, participatory structures that reflect community realities. This effective approach is anchored in four interrelated components: inclusive community engagement, participatory surveillance, localized capacity building, and dynamic feedback mechanisms, all of which inform cross-level policy adaptation. At the foundational Community/Grassroots Level, the diagram illustrates the integration of local communities, farmers, pastoralists, community health workers, local animal health workers (paravets), and environmental custodians. This level is crucial for establishing One Health Community Dialogues (OHCDs) at the kebele or woreda level, institutionalizing inclusive platforms for recurring discussions. These dialogues promote early risk identification, address local health concerns, and facilitate the co-design of prevention strategies, ensuring that direct observations, traditional knowledge, and immediate health concerns (human, animal, environmental) are captured at their source. Examples like Jigjiga University’s pilot activities in pastoralist settings, where nomadic populations contributed to mapping disease hotspots and responding to livestock-related health crises, demonstrate the viability of embedding One Health principles within culturally relevant and mobile-friendly platforms. Ascending to the District/Woreda Level, the diagram illustrates the integration of Woreda Health, Agriculture, and Environmental Protection Offices, along with local veterinary clinics and administrative bodies. This integration is vital for strengthening early detection and real-time reporting through participatory surveillance tools, which can be further enhanced using mobile technology. For instance, the testing of livestock disease tracking apps in Afar and Somali regions allows pastoralists to report outbreaks simultaneously to both veterinary and human health authorities, bridging crucial communication gaps in hard-to-reach areas. Collaboration at this level enables the aggregation and synthesis of diverse, raw information from the grassroots, allowing woreda offices to identify emerging trends, prioritize local issues, and translate community concerns into structured reports, fostering responsiveness in the broader health system and community trust in surveillance. Further up, the Regional Level integrates Regional Health Bureaus, Agriculture Bureaus, Environmental Protection Agencies, and, importantly, Universities & Research Institutions. This is where localized capacity building becomes paramount, involving training community health extension workers and integrating animal health workers and environmental officers into joint capacity development programs. Training modules on risk communication, outbreak preparedness, and participatory learning and action (PLA) techniques empower frontline workers to facilitate dialogue and mediate between sectors. As seen with the African Union-Inter-African Bureau for Animal Resources (AU-IBAR) supported Strengthening Veterinary Governance in Africa project, equipping frontline animal health workers with community mobilization skills and linking them with public health networks reinforces the collaborative core of the One Health model. This integrated analysis ensures that the communication ascending to the national level is not just aggregated data but also includes critical analysis and research insights. Moreover, nurturing “One Health Champions” within communities-trusted figures like teachers, religious leaders, or respected elders, can serve as intermediaries, diffusing information and promoting behavioral change in culturally appropriate ways. Finally, at the National Level, Federal Ministries (Health, Agriculture, Environment, Water), National Research Institutions (EPHI, ILRI), NGOs, international partners, policymakers, and the National One Health Secretariat are integrated. To ensure that communication remains adaptive and responsive, dynamic feedback mechanisms such as community scorecards, suggestion boxes at health posts, participatory monitoring sessions, and community radio broadcasts should be systematized. The example of Ethiopia’s AMR National Action Plan roll-out, where pilot districts integrated feedback from traditional healers and livestock owners into awareness campaigns, demonstrates improved resonance and uptake of public messages. Aligning this comprehensive framework with Ethiopia’s decentralized governance structure and existing Health Extension Program (HEP) offers a practical pathway for institutional sustainability. By embedding these bottom-up communication practices within the standard operations at all levels, the approach can be formalized and scaled through existing service delivery mechanisms. This ensures that insights and data from local levels are not only heard but also acted upon in strategic planning and resource allocation, leading to a more effective, responsive, and sustainable One Health initiative across Ethiopia.

## Hierarchies for bottom-up communication in the one health approach arena

**Figure 1.**
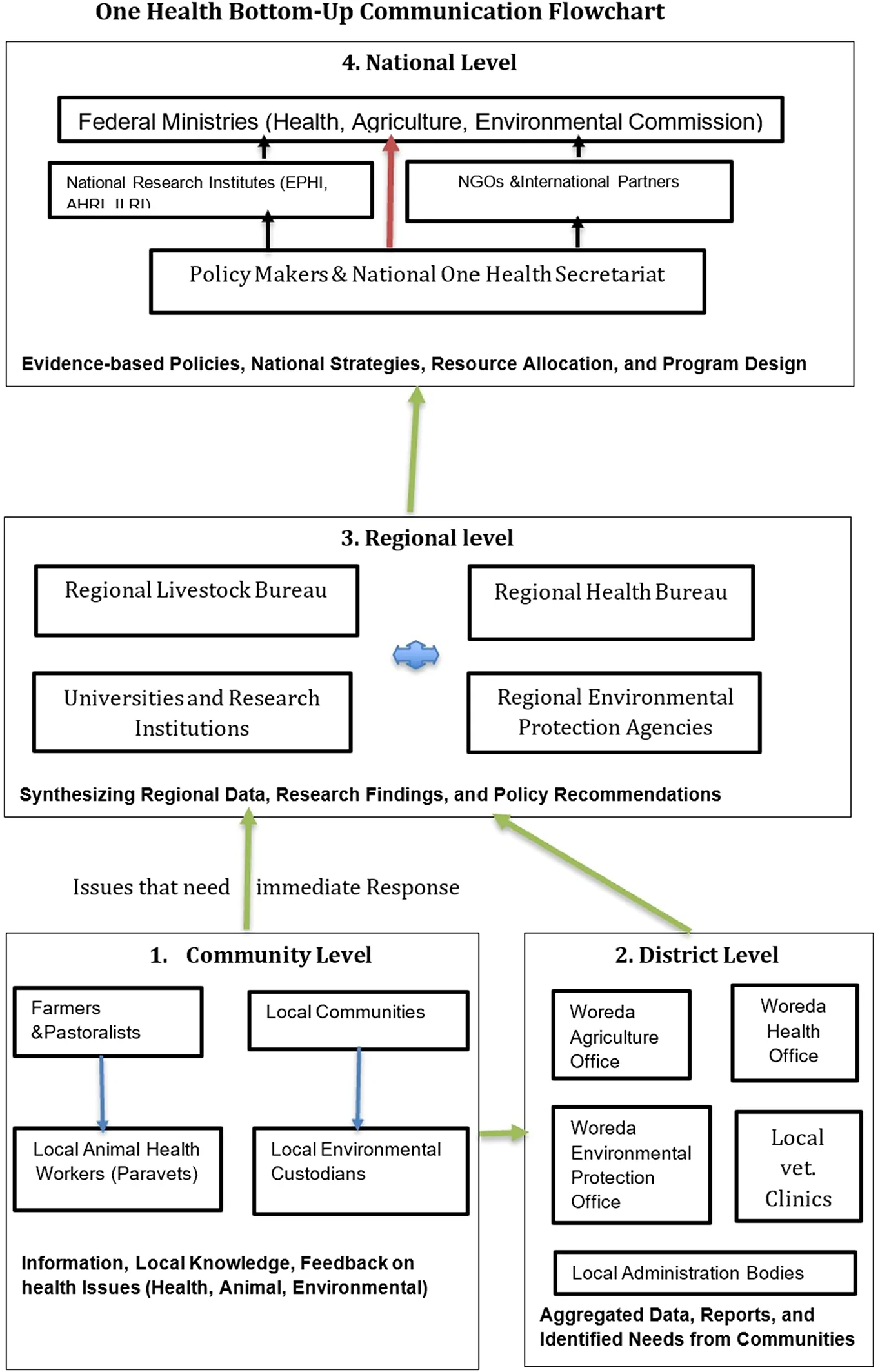
Bottom-up communication flowchart for effective implementation of the one health approach. Source: Designed by the corresponding author (Tadesse Shiferaw, 2025) as part of an original conceptual framework for bottom-up communication in the implementation of the One Health approach in Ethiopia.

The One Health Bottom-Up Communication Flowchart ( Figure 1) illustrates how various stakeholders interact across different levels to ensure the effective implementation of the One Health approach in Ethiopia. At the foundation is the community level, where farmers, pastoralists, and local communities play a vital role by sharing their knowledge, observations, and concerns related to human, animal, and environmental health. They communicate directly with local animal health workers (paravets) and environmental custodians, who serve as immediate links to the formal health and environmental systems. At the district level, offices such as the Woreda Health Office, Agriculture Office, and Environmental Protection Office collect and compile this community-level information. Local veterinary clinics and administration bodies also contribute to identifying and documenting the community’s needs and health issues. These findings are then communicated upward to the regional level.

The regional level acts as a bridge between the local realities and national decision-making. Here, institutions like the Regional Health Bureau, Livestock Bureau, and Environmental Protection Agencies, along with universities and research centers, analyze the data, synthesize research findings, and develop policy recommendations. This ensures that regional insights and challenges are accurately represented at the national level.

At the top, national stakeholders- including federal ministries of Health, Agriculture, and Environment, as well as national research institutes and international partners work through the National One Health Secretariat to design policies, allocate resources, and shape programs. These actions are informed by the data and recommendations received from the lower levels. Overall, the flowchart highlights a participatory, evidence-based communication structure that emphasizes the importance of local engagement in shaping national One Health strategies.

## Conclusion

Bottom-up communication is not just a complementary strategy rather it is a foundational component of effective One Health implementation in Ethiopia. By actively engaging local communities, fostering cross-sectoral collaboration at all administrative levels, and creating responsive feedback systems, Ethiopia can bridge the gap between policy and practice. Institutionalizing this approach through integrated service delivery, legal recognition, and community-driven innovation will be key to building a resilient, inclusive, and sustainable One Health system that addresses both current and emerging health challenges.

### Recommendations

To enhance the implementation and effectiveness of the One Health approach in Ethiopia, it is crucial to institutionalize a bottom-up communication system that emphasizes local engagement, cross-sectoral collaboration, and real-time feedback. This can be accomplished by formally integrating community-based structures, such as One Health Community Dialogues into existing health, agriculture, and environmental platforms at the kebele and woreda levels. Interdisciplinary teams comprising health extension workers, paravets, and environmental officers should be established and trained to lead participatory surveillance and local planning. To support this, Ethiopia should update legal frameworks to mandate and fund multisectoral coordination, improve digital infrastructure in underserved areas, and incentivize community-led innovation. Embedding this model within national systems and decentralization policies will ensure that the One Health initiative is not only inclusive and context-responsive but also sustainable in the long run.

#### Definition of terms

**One health**

An integrated, unifying approach that aims to sustainably balance and optimize the health of humans, animals, plants, and the environment. It recognizes the interconnectedness of all these systems in preventing and controlling health threats.

**Bottom-Up communication**

A participatory communication strategy where information and feedback flow from local communities and frontline actors upward to district, regional, and national decision-makers, ensuring policies and interventions reflect on-the-ground realities.

**Participatory surveillance**

A collaborative method of monitoring health threats, where communities actively contribute observations and reports related to human, animal, and environmental health, often using local knowledge and mobile technologies.

**Intersectoral collaboration**

The coordinated efforts among multiple sectors, such as health, agriculture, and environment, to work together toward shared public health goals, particularly in addressing complex challenges like zoonotic diseases or antimicrobial resistance.

**Decentralized governance**

A system where decision-making authority and resources are distributed from the national level to regional, district, and community levels, enabling localized planning and implementation of public services.

**Community engagement**

The process involves actively involving local individuals, leaders, and organizations in identifying health priorities, designing interventions, and evaluating outcomes to ensure relevance, ownership, and sustainability.

**Zoonotic diseases**

Diseases that can be transmitted between animals and humans, such as rabies, anthrax, and brucellosis, are prevalent in livestock-dependent communities in Ethiopia.

**Feedback mechanisms**

Structured systems, such as community scorecards, suggestion boxes, and local forums, that allow communities to provide input, assess services, and influence policy or program adjustments.

**Health Extension Program (HEP)**

Ethiopia’s flagship community-based health delivery strategy deploys health extension workers to provide essential health services and education at the grassroots level.

**Community Health Workers (CHWs)**

Locally recruited and trained individuals who provide basic health services, promote healthy behaviors, and act as a bridge between communities and formal health systems.

**Ethical approval and consent to participate**

This study did not involve the collection of primary data from human or animal subjects. It is based entirely on a review and synthesis of publicly available literature, including published articles, national and international guidelines, One Health strategy documents, government frameworks, and general communication science. Therefore, ethical approval and participant consent were not required.

## Consent for publication

Consent for publication was not applicable as this study does not contain identifiable patient data.

## Data Availability

No new datasets were generated or analyzed in this study. This article relies entirely on reviewing and synthesizing publicly available literature, policy documents, and institutional guidelines. All referenced sources are openly accessible and properly cited within the manuscript. Consequently, there is no original data associated with this article that needs to be stored in a data repository.
