# Bottom-Up Communication Approach for Effective Implementation of the One Health Initiative in Ethiopia

**DOI:** 10.12688/f1000research.166948.2

**Published:** 2026-04-06

**Authors:** Tadesse Shiferaw Chekol, Samuel Degie Abebe, Metasebia Tegegn Baheru

**Affiliations:** 1 One Health, Armauer Hansen Research Institute, Addis Ababa, Addis Ababa, 1005, Ethiopia; 2 Health Information and Counseling, Federal Ministry of Health, Addis Ababa, Addis Ababa, 1234, Ethiopia; 3 Health Science, Addis Ababa University College of Health Sciences, Addis Ababa, Addis Ababa, 9086, Ethiopia

**Keywords:** One Health, Bottom-up Communication, Ethiopia

## Abstract

The One Health approach, which integrates human, animal, environmental, and plant health, is Crucial for addressing complex public health challenges in Ethiopia. It addresses zoonotic diseases, antimicrobial resistance, and ecological degradation. These issues have increased the national burden alongside efforts for infectious disease prevention and control. However, effective implementation relies on communication strategies that are participatory, context-specific, and responsive to local realities. This paper presents a bottom-up communication framework designed to enhance local ownership, strengthen multisectoral collaboration, and align policy with community needs. The proposed approach emphasizes inclusive stakeholder engagement, the capacity-building of frontline actors, and the establishment of continuous feedback loops connecting community, district, and national levels to ensure the adaptive and sustainable implementation of One Health in Ethiopia.

## Introduction

One Health is an integrated and unifying approach that aims to sustainably balance and optimize the health of humans, animals, plants, and ecosystems. It recognizes that the health of humans, domestic and wild animals, plants, and the broader environment (including ecosystems) are closely linked and interdependent. ^1^ Ethiopia faces a complex array of interconnected health challenges that underscore the urgent need for a robust One Health approach. The country continues to struggle with endemic zoonotic diseases such as rabies, anthrax, and brucellosis, along with periodic outbreaks of emerging infectious diseases like Rift Valley fever and avian influenza. The growing burden of antimicrobial resistance (AMR), exacerbated by the unregulated use of antibiotics in both the human and animal health sectors, is another one health issue. The country has prioritized five zoonotic diseases: rabies, anthrax, brucellosis, leptospirosis, and echinococcosis. ^2^ Currently, Mpox (formerly known as monkeypox) has re-emerged as a global public health concern since 2022, with outbreaks reported across multiple regions. Although Ethiopia has reported limited cases, the international spread of the disease highlights the importance of strengthening surveillance systems and cross-sectoral communication mechanisms capable of rapidly detecting and responding to emerging zoonotic threats. ^3^ Currently, Mpox is another newly emerging health threat that requires special attention. These issues are further exacerbated by rapid population growth, environmental degradation, and high levels of human-animal-environment interaction, particularly in pastoral and agropastoral communities, where livelihoods heavily depend on livestock and natural resources. In recognition of these threats, Ethiopia has made significant strides in developing national-level frameworks to institutionalize the One Health approach. This includes establishing the One Health Steering Committee, formulating the One Health Strategic Plan (2022–2026), and fostering cross-sectoral collaboration among the Ministries of Health, the Ministry of Agriculture, and the Environment Commission. ^4^ However, the practical implementation of these frameworks remains uneven and often limited in reach. One of the primary barriers is the dominance of top-down communication structures that prioritize centralized decision-making and technical directives while overlooking local knowledge, cultural dynamics, and the practical realities of frontline actors. In Ethiopia, disease signals detected at the community level by health extension workers or animal health professionals are frequently transmitted through several administrative levels before reaching regional or national coordination platforms. Such reporting hierarchies may delay the recognition of zoonotic threats and limit opportunities for rapid multisectoral response. Strengthening communication pathways that facilitate the upward flow of information from community actors may therefore enhance the responsiveness of One Health governance systems. ^5^ Research and global experiences increasingly demonstrate that participatory, bottom-up communication strategies are critical to the success of One Health initiatives. These strategies help build trust between communities and institutions, encourage meaningful behavioral change, and facilitate coordination among sectors that have historically operated in silos. ^6, 7^ Community-based health surveillance that integrates human and animal health has shown promise in Ethiopia’s remote pastoral areas. Studies in the Somali Regional State demonstrated that engaging local leaders and community health workers in syndromic surveillance, supported by mobile technology, improves early detection and reporting of human and animal health events. The One Health Surveillance and Response System (OHSRS) approach, involving multiple sectors and community participation, enhanced zoonotic disease detection and response. ^8^ Community-based animal health workers (CAHWs) have been crucial in delivering veterinary services in remote areas, though sustainability remains a challenge. ^9^ The integration of priority zoonotic disease surveillance into existing polio eradication programs has shown success, with community volunteers reporting disease alerts and reaching numerous households. Collectively, these experiences suggest that community-level engagement can play a critical role in strengthening surveillance, improving communication between sectors, and enabling more responsive health governance systems. Lessons from community-based surveillance initiatives indicate that integrating local observations with institutional reporting mechanisms can enhance early detection and support more coordinated responses to emerging health risks. ^10^ However, challenges such as delayed integrated monitoring mechanisms persist. ^11^ These initiatives demonstrate the potential of community-based approaches in strengthening local health surveillance and response systems. This paper, therefore, explores the rationale for a bottom-up communication approach and proposes a practical framework for its integration into Ethiopia’s One Health system. Bottom-up communication approaches are closely aligned with participatory governance and decentralized health system frameworks, which emphasize the role of local actors in shaping policy implementation and decision-making processes. These approaches recognize that community members and frontline workers often possess contextual knowledge that can improve the detection of emerging health threats and strengthen the responsiveness of surveillance and response systems. ^12^

Despite the establishment of national One Health coordination mechanisms in Ethiopia, communication pathways linking community-level actors with district, regional, and national decision-making structures remain insufficiently institutionalized. Observations from communities, health extension workers, and animal health actors are often transmitted through multiple administrative layers before reaching higher-level coordination bodies, which may delay the detection of emerging health threats and limit the timeliness of multisectoral responses. These communication gaps are particularly relevant in settings where close interactions between humans, livestock, and the environment increase the risk of zoonotic disease emergence. Strengthening mechanisms that enable the timely transmission of community-level observations to formal governance structures is therefore critical for effective One Health implementation. ^13^ This article presents a conceptual and policy-oriented analysis informed by a narrative review of relevant literature, national policy documents, and implementation experiences related to One Health initiatives in Ethiopia. The objective is to synthesize existing knowledge and propose a structured framework for strengthening bottom-up communication within Ethiopia’s multisectoral health governance system. ^14^

## Approach to literature identification

This article draws on a narrative review of literature and policy documents related to One Health implementation and health communication systems. Relevant sources were identified through searches of academic databases, including PubMed and Google Scholar, as well as through review of institutional publications produced by global and national One Health stakeholders. Particular attention was given to national policy frameworks developed in Ethiopia, including the national One Health Strategic Plan and multisectoral coordination mechanisms. International frameworks such as the One Health Joint Plan of Action were also consulted to contextualize the proposed communication model. The objective of this approach was not to conduct a systematic review but rather to synthesize policy experiences, conceptual frameworks, and operational examples that can inform the development of a bottom-up communication framework for One Health implementation. ^15^

## The rationale for bottom-up communication in one health

Communication is defined as the structured process of exchanging information to achieve shared understanding and coordinated action. It plays a pivotal role in enabling cross-sector collaboration and community engagement within One Health systems. It is acknowledged not only as an operational necessity but also as a strategic enabler of coordination, engagement, learning, and adaptive response in multisectoral One Health implementation. ^1^ Bottom-up communication strategies are increasingly seen as essential for the successful implementation of complex policy frameworks such as the One Health initiative, particularly in decentralized and multisectoral contexts like Ethiopia. This approach empowers local stakeholders by incorporating their lived experiences, knowledge systems, and cultural contexts into decision-making processes, thereby enhancing the relevance, acceptability, and sustainability of interventions. In rural Ethiopian communities, where zoonotic diseases frequently emerge, local actors are often the first to observe and respond to health threats; yet, they remain underrepresented in formal surveillance systems and policy dialogues. ^16^ In Ethiopia, community-level actors such as health extension workers, animal health professionals, and local leaders frequently play an important role in detecting early signals of zoonotic or environmental health risks. However, these observations are not always systematically integrated into formal surveillance systems. Evidence from community-based surveillance initiatives suggests that incorporating local knowledge and frontline observations into institutional reporting structures can strengthen early detection and improve coordination across the human health, animal health, and environmental sectors. ^17^ Bottom-up communication cultivates trust in institutions and scientific initiatives, which is critical for effective risk communication and compliance during public health emergencies. ^18^ Evidence from public health emergencies has shown that trust between communities and health authorities plays a critical role in improving adherence to public health recommendations and facilitating timely reporting of health threats. Communication strategies that incorporate local engagement and dialogue mechanisms have been shown to strengthen trust and improve risk communication during health emergencies ^19^ It also supports innovative and context-specific solutions by fostering continuous feedback, learning, and problem-solving among community members, health workers, and government actors. Furthermore, this approach facilitates both horizontal collaboration among sectors (such as health, agriculture, and environment) and vertical integration across administrative levels, from the community up to national policymaking bodies. Without inclusive communication mechanisms, implementation efforts risk becoming top-down directives that fail to resonate with or be adopted by the very populations they aim to serve. Therefore, bottom-up communication is not merely an engagement tool but a strategic necessity for realizing the full potential of the One Health approach in Ethiopia.

Several structural barriers contribute to the limited participation of local actors in formal surveillance systems. These include hierarchical reporting structures, limited digital reporting tools at the community level, and institutional fragmentation across sectors responsible for human, animal, and environmental health. Addressing these barriers requires communication mechanisms that facilitate more direct engagement between community actors and multisectoral coordination platforms while strengthening feedback loops between administrative levels. ^20^

## Equity and inclusive participation

Effective bottom-up communication within One Health systems must account for social, gender, and cultural dynamics that shape participation in community decision-making processes. In many pastoral and rural contexts, access to information and influence over community discussions may vary significantly by gender, age, and social status. Women, youth, and marginalized pastoral groups may face barriers related to literacy, language, mobility, or local power hierarchies. To ensure equitable participation, One Health Community Dialogues should incorporate inclusive facilitation approaches, including representation quotas where appropriate, use of local languages, and participatory methods adapted for low-literacy populations. Training facilitators to recognize and mitigate local power imbalances can further help ensure that diverse perspectives are represented. Integrating such equity-focused approaches strengthens the legitimacy and effectiveness of community-driven surveillance and response systems. ^21^

## Institutional considerations and policy alignment

Effective implementation of a bottom-up communication approach in Ethiopia’s One Health framework requires institutional coordination, decentralization, and alignment with existing service delivery systems. While the national One Health Steering Committee provides strategic direction, weak linkages between federal, regional, and community levels limit the flow of local insights into national planning. ^22^ Integrating One Health into Ethiopia’s decentralized governance system, particularly through health, agriculture, and environmental offices at the woreda level, is essential. These coordination challenges are often influenced by administrative mandates that assign responsibilities to separate institutions, differences in sectoral priorities, and limited financial mechanisms for joint planning across ministries and regional bureaus. Such institutional fragmentation can make it difficult to operationalize multisectoral approaches without clear coordination structures and shared accountability mechanisms. ^23^

A practical entry point is the Health Extension Program (HEP), which has a wide community presence but operates largely in isolation from veterinary and environmental services. ^24^ Establishing interdisciplinary teams, bringing together health extension workers, animal health assistants, and environmental officers, can strengthen collaboration at the grassroots. Initiatives such as the Oromia rabies control program have shown that coordinated community-level responses improve both efficiency and trust. While the Health Extension Program provides an important platform for community engagement in Ethiopia, health extension workers often face heavy workloads and multiple programmatic responsibilities. Additional training, supportive supervision, and improved coordination mechanisms may therefore be required to enable them to effectively contribute to One Health communication and surveillance activities. ^25^

Policy alignment is also critical. While Ethiopia’s One Health Strategic Plan (2022–2026) outlines multisectoral goals, operational gaps remain due to fragmented data systems, siloed budgets, and unclear mandates. ^26^ Strengthening joint planning and reporting mechanisms, particularly at regional and district levels, can enhance coordination. Universities and research institutions can play a key role here, as seen in projects like the One Health data integration pilot at Jimma University.

To institutionalize this approach, existing legal and regulatory frameworks should be updated to formally recognize intersectoral collaboration. Empowering regional bureaus with the authority and resources to coordinate locally appropriate One Health actions, especially in high-risk border areas, will improve responsiveness. Finally, creating incentives for community-level innovation and feedback, such as flexible budgets and participatory monitoring tools, can reinforce local ownership and sustain the bottom-up model. Multisectoral coordination teams could be established at the woreda level within existing administrative structures. Clear reporting lines linking these teams to regional One Health coordination bodies would help facilitate information sharing and strengthen accountability across administrative levels. ^27^

## Challenges and mitigation strategies

For analytical clarity, challenges affecting bottom-up communication in the One Health system can be broadly categorized into structural constraints, operational constraints, and resource-related constraints. Structural constraints include governance fragmentation and policy misalignment across sectors. Operational constraints relate to workforce capacity, coordination mechanisms, and communication processes. Resource-related constraints include infrastructure limitations, technological gaps, and financial constraints affecting surveillance and reporting systems. ^28^ Implementing a bottom-up communication approach within Ethiopia’s One Health framework faces several practical challenges. First, limited infrastructure, such as poor internet access, inadequate transportation, and weak mobile networks, hinders timely information flow between communities and institutions, particularly in remote pastoral areas. Limited digital infrastructure and shortages of trained personnel in remote and pastoral areas can further constrain the effectiveness of surveillance systems and delay the transmission of critical information to higher-level coordination platforms. Strengthening digital reporting tools and expanding training opportunities for frontline workers may therefore support more timely information exchange. ^29^ Second, human resource gaps exist at the community level, where health and veterinary workers often lack cross-sectoral training and coordination mechanisms. Additionally, vertical and horizontal fragmentation across ministries leads to parallel systems, duplicative efforts, and policy misalignment.

Another significant barrier is the lack of clear legal mandates for multisectoral coordination, which reduces accountability and limits the formal recognition of local actors’ contributions. Inconsistent data sharing and weak surveillance integration across human, animal, and environmental health systems also delay early detection and response efforts. Lastly, community engagement remains underdeveloped, with limited structures for incorporating grassroots perspectives into planning and feedback loops. ^30^

To mitigate these challenges, targeted investments should prioritize expanding digital infrastructure in underserved regions and strengthening the capacity of frontline workers through interdisciplinary training. Embedding One Health teams within existing structures, such as health posts and woreda offices, can improve integration and resource sharing. Updating national laws and operational guidelines to formalize cross-sectoral collaboration will provide institutional clarity and enable better coordination. At the community level, introducing low-cost, culturally appropriate communication tools (e.g., local radio, mobile alerts, and participatory forums) can enhance information flow and foster engagement. Ultimately, addressing these challenges will require a deliberate, coordinated effort to bridge system-level gaps while empowering local actors to lead context-specific solutions. Implementing these strategies will require a phased approach that considers available resources, institutional capacity, and regional differences. Pilot initiatives and incremental expansion may help ensure sustainability while allowing lessons learned from early implementation to inform broader scaling of bottom-up communication mechanisms. ^31^

## Bottom-Up communication framework

The proposed bottom-up communication framework operates across four governance levels: community, district (woreda), regional, and national. Each level plays a complementary role in detecting, communicating, and responding to health threats while maintaining feedback mechanisms that ensure community-level information informs higher-level decision-making processes. ^32^ The integrated bottom-up communication model for One Health in Ethiopia is designed to move beyond abstract coordination by embracing grounded, participatory structures that reflect community realities. This effective approach is anchored in four interrelated components: inclusive community engagement, participatory surveillance, localized capacity building, and dynamic feedback mechanisms, all of which inform cross-level policy adaptation. At the foundational Community/Grassroots Level, the diagram illustrates the integration of local communities, farmers, pastoralists, community health workers, local animal health workers (paravets), and environmental custodians. This level is crucial for establishing One Health Community Dialogues (OHCDs) at the kebele or woreda level, institutionalizing inclusive platforms for recurring discussions. These dialogues promote early risk identification, address local health concerns, and facilitate the co-design of prevention strategies, ensuring that direct observations, traditional knowledge, and immediate health concerns (human, animal, environmental) are captured at their source. Examples like Jigjiga University’s pilot activities in pastoralist settings, where nomadic populations contributed to mapping disease hotspots and responding to livestock-related health crises, demonstrate the viability of embedding One Health principles within culturally relevant and mobile-friendly platforms. Ascending to the District/Woreda Level, the diagram illustrates the integration of Woreda Health, Agriculture, and Environmental Protection Offices, along with local veterinary clinics and administrative bodies. This integration is vital for strengthening early detection and real-time reporting through participatory surveillance tools, which can be further enhanced using mobile technology. For instance, the testing of livestock disease tracking apps in Afar and Somali regions allows pastoralists to report outbreaks simultaneously to both veterinary and human health authorities, bridging crucial communication gaps in hard-to-reach areas. Collaboration at this level enables the aggregation and synthesis of diverse, raw information from the grassroots, allowing woreda offices to identify emerging trends, prioritize local issues, and translate community concerns into structured reports, fostering responsiveness in the broader health system and community trust in surveillance. Further up, the Regional Level integrates Regional Health Bureaus, Agriculture Bureaus, Environmental Protection Agencies, and, importantly, Universities & Research Institutions. This is where localized capacity building becomes paramount, involving training community health extension workers and integrating animal health workers and environmental officers into joint capacity development programs. Training modules on risk communication, outbreak preparedness, and participatory learning and action (PLA) techniques empower frontline workers to facilitate dialogue and mediate between sectors. As seen with the African Union-Inter-African Bureau for Animal Resources (AU-IBAR) supported Strengthening Veterinary Governance in Africa project, equipping frontline animal health workers with community mobilization skills and linking them with public health networks reinforces the collaborative core of the One Health model. This integrated analysis ensures that the communication ascending to the national level is not just aggregated data but also includes critical analysis and research insights. Moreover, nurturing “One Health Champions” within communities-trusted figures like teachers, religious leaders, or respected elders, can serve as intermediaries, diffusing information and promoting behavioral change in culturally appropriate ways. Finally, at the National Level, Federal Ministries (Health, Agriculture, Environment, Water), National Research Institutions (EPHI, ILRI), NGOs, international partners, policymakers, and the National One Health Secretariat are integrated. To ensure that communication remains adaptive and responsive, dynamic feedback mechanisms such as community scorecards, suggestion boxes at health posts, participatory monitoring sessions, and community radio broadcasts should be systematized. The example of Ethiopia’s AMR National Action Plan roll-out, where pilot districts integrated feedback from traditional healers and livestock owners into awareness campaigns, demonstrates improved resonance and uptake of public messages. Aligning this comprehensive framework with Ethiopia’s decentralized governance structure and existing Health Extension Program (HEP) offers a practical pathway for institutional sustainability. By embedding these bottom-up communication practices within the standard operations at all levels, the approach can be formalized and scaled through existing service delivery mechanisms. This ensures that insights and data from local levels are not only heard but also acted upon in strategic planning and resource allocation, leading to a more effective, responsive, and sustainable One Health initiative across Ethiopia.

**Table 1.** Proposed bottom-up communication framework for one health implementation.

Framework component	Description	Key actors	Example indicators
Inclusive community engagement	One Health Community Dialogues	Community leaders, health extension workers, pastoral representatives	Number of community dialogues conducted; diversity of participants
Participatory Surveillance	Community-based disease reporting	HEWs, animal, health workers, community volunteers	Timeliness of reporting; number of comunity alerts
Localized capacity building	Training and mentoring of frontline actors	Woreda health and livestock offices	Number of trained personnel; communication skill assessments
Dynamic feedback mechanisms	Vertical and horizontal communication channels	Woreda coordination platforms	Response time to community alerts; feedback loops established

## Hierarchies for bottom-up communication in the one health approach arena

The One Health Bottom-Up Communication Flowchart ( Figure 1) illustrates how various stakeholders interact across different levels to ensure the effective implementation of the One Health approach in Ethiopia. At the foundation is the community level, where farmers, pastoralists, and local communities play a vital role by sharing their knowledge, observations, and concerns related to human, animal, and environmental health. They communicate directly with local animal health workers (paravets) and environmental custodians, who serve as immediate links to the formal health and environmental systems. At the district level, offices such as the Woreda Health Office, Agriculture Office, and Environmental Protection Office collect and compile this community-level information. Local veterinary clinics and administration bodies also contribute to identifying and documenting the community’s needs and health issues. These findings are then communicated upward to the regional level.

**Figure 1.**
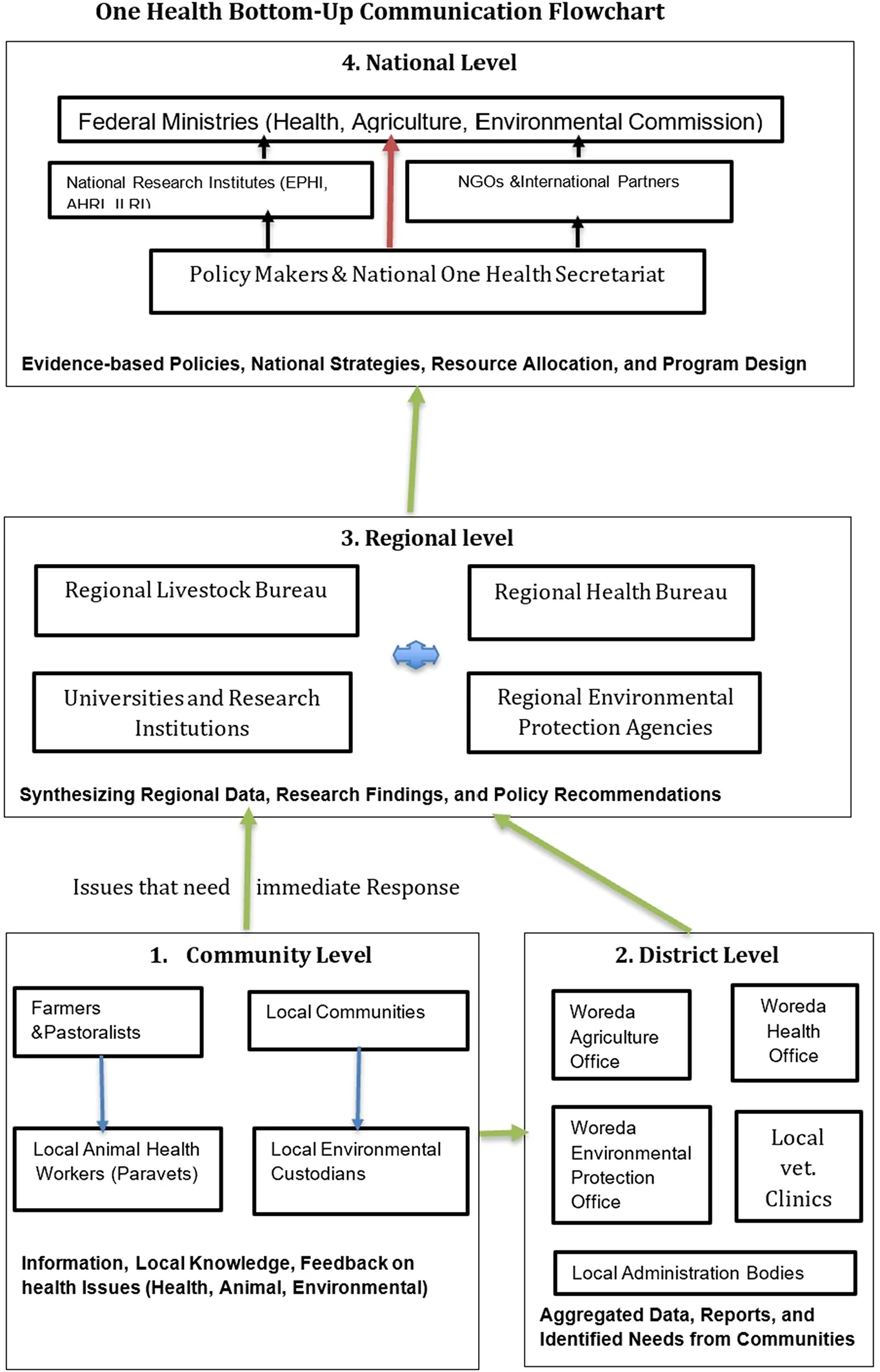
Bottom-up communication flowchart for effective implementation of the one health approach. Source: Designed by the corresponding author (Tadesse Shiferaw, 2025) as part of an original conceptual framework for bottom-up communication in the implementation of the One Health approach in Ethiopia.

The regional level acts as a bridge between the local realities and national decision-making. Here, institutions like the Regional Health Bureau, Livestock Bureau, and Environmental Protection Agencies, along with universities and research centers, analyze the data, synthesize research findings, and develop policy recommendations. This ensures that regional insights and challenges are accurately represented at the national level.

At the top, national stakeholders- including federal ministries of Health, Agriculture, and Environment, as well as national research institutes and international partners work through the National One Health Secretariat to design policies, allocate resources, and shape programs. These actions are informed by the data and recommendations received from the lower levels. Overall, the flowchart highlights a participatory, evidence-based communication structure that emphasizes the importance of local engagement in shaping national One Health strategies. Effective implementation of bottom-up communication mechanisms will depend on the presence of clear feedback loops between administrative levels. Ensuring that information shared by regional and community actors informs national decision-making processes can strengthen accountability and improve the responsiveness of multisectoral health systems. ^33^

## Limitation

This article presents a conceptual framework intended to guide the strengthening of bottom-up communication within Ethiopia’s One Health system. The framework is based primarily on the synthesis of policy experiences, programmatic examples, and existing literature rather than empirical evaluation. As such, the effectiveness of the proposed communication model has not yet been systematically tested across diverse operational settings. In addition, the examples discussed in this paper rely partly on programmatic reports and illustrative initiatives that may not fully capture implementation variability across regions. Future research should therefore empirically assess the feasibility, effectiveness, and sustainability of bottom-up communication mechanisms within One Health governance structures, including evaluation of community engagement processes, data flows, and health outcomes. ^34^

## Conclusion

Bottom-up communication should be recognized as a core component of effective One Health implementation in Ethiopia. Actively engaging local communities is essential. Strengthening cross-sectoral collaboration across administrative levels is also critical. Responsive feedback mechanisms can further help connect local realities with national priorities. Together, these efforts can help bridge the persistent gap between policy development and implementation on the ground. This article proposes a conceptual framework to strengthen communication within Ethiopia’s evolving One Health system. The framework emphasizes community engagement, participatory surveillance, localized capacity development, and continuous information exchange between communities and institutional governance structures. These approaches can support more responsive and context-sensitive decision-making.

Institutionalizing such practices will be important for long-term progress. This may include integrating services across sectors, strengthening legal recognition of collaborative mechanisms, and encouraging community-driven innovation. These efforts could contribute to building a more resilient, inclusive, and sustainable One Health system that is better prepared to address current and emerging health challenges. However, further empirical research and implementation studies are needed. Future work should assess how these mechanisms function in practice and refine operational strategies across Ethiopia’s diverse sociocultural and institutional contexts.

### Recommendations

To strengthen the implementation and effectiveness of the One Health approach in Ethiopia, it is important to institutionalize a bottom-up communication system that promotes community engagement, cross-sectoral collaboration, and continuous feedback between local and national governance levels. Such a system should be embedded within existing health, agriculture, and environmental coordination structures while strengthening participatory surveillance and multisectoral planning.

#### Short-term actions

• Strengthen community engagement platforms by integrating structured One Health Community Dialogues within existing kebele- and woreda-level health, agriculture, and environmental programs.
• Improve coordination among frontline actors by establishing interdisciplinary collaboration between health extension workers, veterinary professionals, and environmental officers to support participatory surveillance and local planning.

#### Medium-term actions

• Expand participatory surveillance systems that incorporate community-based reporting mechanisms and local knowledge into formal surveillance structures.
• Strengthen multisectoral training and capacity-building programs for frontline human, animal, and environmental health professionals to support integrated risk detection and response.

#### Long-term actions

• Institutionalize bottom-up communication mechanisms within national and regional One Health governance structures through updated legal and policy frameworks that mandate multisectoral coordination and sustainable financing.
• Strengthen monitoring, evaluation, and learning systems, improve digital reporting infrastructure in underserved areas, and incentivize community-led innovation to ensure the long-term sustainability and scalability of bottom-up One Health communication systems.

#### Definition of terms

**One health**

An integrated, unifying approach that aims to sustainably balance and optimize the health of humans, animals, plants, and the environment. It recognizes the interconnectedness of all these systems in preventing and controlling health threats.

**Bottom-Up communication**

A participatory communication strategy where information and feedback flow from local communities and frontline actors upward to district, regional, and national decision-makers, ensuring policies and interventions reflect on-the-ground realities.

**Participatory surveillance**

A collaborative method of monitoring health threats, where communities actively contribute observations and reports related to human, animal, and environmental health, often using local knowledge and mobile technologies.

**Intersectoral collaboration**

The coordinated efforts among multiple sectors, such as health, agriculture, and environment, to work together toward shared public health goals, particularly in addressing complex challenges like zoonotic diseases or antimicrobial resistance.

**Decentralized governance**

A system where decision-making authority and resources are distributed from the national level to regional, district, and community levels, enabling localized planning and implementation of public services.

**Community engagement**

The process involves actively involving local individuals, leaders, and organizations in identifying health priorities, designing interventions, and evaluating outcomes to ensure relevance, ownership, and sustainability.

**Zoonotic diseases**

Diseases that can be transmitted between animals and humans, such as rabies, anthrax, and brucellosis, are prevalent in livestock-dependent communities in Ethiopia.

**Feedback mechanisms**

Structured systems, such as community scorecards, suggestion boxes, and local forums, that allow communities to provide input, assess services, and influence policy or program adjustments.

**Health Extension Program (HEP)**

Ethiopia’s flagship community-based health delivery strategy deploys health extension workers to provide essential health services and education at the grassroots level.

**Community Health Workers (CHWs)**

Locally recruited and trained individuals who provide basic health services, promote healthy behaviors, and act as a bridge between communities and formal health systems.

**Ethical approval and consent to participate**

This study did not involve the collection of primary data from human or animal subjects. It is based entirely on a review and synthesis of publicly available literature, including published articles, national and international guidelines, One Health strategy documents, government frameworks, and general communication science. Therefore, ethical approval and participant consent were not required.

## Consent for publication

Consent for publication was not applicable as this study does not contain identifiable patient data.

## Data Availability

No new datasets were generated or analyzed in this study. This article relies entirely on reviewing and synthesizing publicly available literature, policy documents, and institutional guidelines. All referenced sources are openly accessible and properly cited within the manuscript. Consequently, there is no original data associated with this article that needs to be stored in a data repository.
