# Supplementary material for: Bottom-Up Communication Approach for Effective Implementation of the One Health Initiative in Ethiopia
Source: F1000Res. 2026 Apr 6;14:866. Originally published 2025 Sep 3. [Version 2] doi: 10.12688/f1000research.166948.2 (PMC13560016; doi:10.12688/f1000research.166948.2)
Supplement: Peer review history

# Reviewer report: Reviewer response for version 2

**DOI:** 10.5256/f1000research.197557.r510071

**Published:** 2026-09-02

**Authors:** Andrea Haekyung Haselbeck

**Affiliations:** 1 The University of Edinburgh, Edinburgh, Scotland, UK

This review is a conceptual article arguing that Ethiopia's One Helath system is limited by its top to down communication run. The authors propose a four-tier bottom up communication framework built on inclusive community engagement, participatory surveillance, capacity building and dynamic feedback mechanisms. 

 The introduction states that signals "are frequently transmitted through several administrative levels before reaching regional or national coordination platforms" and that "such reporting hierarchies may delay the recognition of zoonotic threats." The remedy the authors are offering includes four levels, i.e. community to district to regional to national.  Sequential aggregation at each. That is the existing reporting chain with participatory vocabulary applied to it. The framework does not shorten a pathway, remove a layer, or let's say create a direct channel from a kebele to the National One Health Secretariat. The flowchart described in Figure 1 text is a chart of Ethiopia's current administrative structure with arrows pointing up. The author cite Arnstein's ladder of citizen participation. But Arnstiens entire argument is that participation without redistribution of power is tokenism. By the standard of the framework the author invoke, this sits on exactly the tokenism rungs. As a consequence, the authors cite Arnstein without really applying it. Overall, there seems to be an issue with the citations. Examples provided throughout the manuscript, such as Jigjiga University's pilot activities with nomadic populations; livestock disease tracking apps tested in Afar and Somali; the Oromia rabies control program; the One Health data integration pilot at Jimma University; AMR National Action Plan pilot districts integrating traditional healer feedback, are not backed with any citation. Further MPox cases are mentioned, but undated and only a generic WHO situation update provided, outbreaks of emerging infectious diseases also mentioned but not citated. References 2, 6, 10 seem to refer to the wrong article, as the content does not connect to the context it is aimed at supporting. 

 A major question that remains it that IDSR is already implemented in Ethiopia, but it does not seem to work, if the authors claim that this angle needs to be addressed. Then, why is it not working and how would this framework suggested here change the issue specifically? 

 The article falls short on the analysis and conclusion to demonstrate that the One Health architecture in Ethiopia is stronger on paper than in practice. It is worth indexing on an important issue as described here, that frontline observations do not reach coordinating bodies fast enough and so then does response to these.

Is the review written in accessible language?

Yes

Are all factual statements correct and adequately supported by citations?

Partly

Are the conclusions drawn appropriate in the context of the current research literature?

Partly

Is the topic of the review discussed comprehensively in the context of the current literature?

Yes

Reviewer Expertise:

Infectious disease surveillance; public health policy; vaccine introduction; health capacity building

I confirm that I have read this submission and believe that I have an appropriate level of expertise to confirm that it is of an acceptable scientific standard, however I have significant reservations, as outlined above.

# Reviewer report: Reviewer response for version 2

**DOI:** 10.5256/f1000research.197557.r495891

**Published:** 2026-08-18

**Authors:** Noore Alam

**Affiliations:** 1 Queensland Health, Brisbane, Australia

**General comments**

 This review addresses an important and timely topic and makes a useful contribution by highlighting the role of bottom-up communication in strengthening One Health implementation in Ethiopia. Its focus on community engagement, participatory surveillance, inclusive governance, and cross-level feedback mechanisms is particularly relevant in the Ethiopian context.

 As a review article, the manuscript would be strengthened by greater clarity regarding the scope of the review, the approach used to identify and select the literature and policy sources, and the specific contribution of the proposed framework in relation to the existing body of knowledge. While the narrative and policy-oriented nature of the article is appropriate, several sections are repetitive, and some claims would benefit from stronger referencing or more cautious wording. The proposed framework would also be strengthened by a clearer explanation of how it was derived from the reviewed literature and policy experiences.

 Overall, this is a useful and relevant review on an important topic. It makes a worthwhile contribution by drawing attention to the importance of bottom-up communication in One Health implementation in Ethiopia. With some revisions to improve clarity of scope, transparency of the review approach, synthesis of the literature, and grounding of the proposed framework, the article would be further strengthened.

Is the review written in accessible language?

Yes

Are all factual statements correct and adequately supported by citations?

Partly

Are the conclusions drawn appropriate in the context of the current research literature?

Yes

Is the topic of the review discussed comprehensively in the context of the current literature?

Partly

Reviewer Expertise:

Epidemiology, One Health, Emerging Infectious Diseases.

I confirm that I have read this submission and believe that I have an appropriate level of expertise to confirm that it is of an acceptable scientific standard.

# Response

**Published:** 2026-08-19

**Authors:** Tadesse Shiferaw Chekol

**Affiliations:** One Health Research Division, Armauer Hansen Research Institute, Addis Ababa, Addis Ababa, Ethiopia

We sincerely thank the reviewer for the careful and constructive assessment of our manuscript. We appreciate the recognition of the relevance and contribution of the review, particularly its focus on bottom-up communication, community engagement, participatory surveillance, inclusive governance, and cross-level feedback mechanisms in strengthening One Health implementation in Ethiopia.

1. We have carefully considered the major comments and plan to revise the manuscript accordingly. The planned revisions will focus on the following key areas:
2. We will clarify the scope and nature of the review and provide a more transparent description of how relevant literature, policy documents, and implementation experiences were identified and selected.
3. We will strengthen the synthesis of the reviewed literature and policy experiences, with greater emphasis on how the available evidence supports the rationale for bottom-up communication in the Ethiopian One Health context.
4. We will review factual statements throughout the manuscript and strengthen citation support where needed. We will also use more cautious wording where the available evidence does not support definitive claims.
5. We will provide a clearer explanation of how the proposed bottom-up communication framework was derived from the reviewed literature, policy documents, and implementation experiences. We will strengthen the links between the evidence reviewed and the four core components of the framework.
6. We will review the manuscript for repetitive content and improve the organization and synthesis of the discussion to make the main argument more concise and coherent.

We will further clarify the practical relevance of the framework, including its relationship with existing One Health governance structures and its potential application across community, district, regional, and national levels.

 We thank the reviewer again for these valuable comments. We believe these revisions will improve the clarity, transparency, evidence base, and practical relevance of the manuscript while maintaining its central contribution on bottom-up communication for One Health implementation in Ethiopia.

# Reviewer report: Reviewer response for version 2

**DOI:** 10.5256/f1000research.197557.r473234

**Published:** 2026-04-17

**Authors:** Chinenyenwa M. D. Ohia

**Affiliations:** 1 Biosciences, Indian Institute of Technology Bombay (Ringgold ID: 29491), Mumbai, Maharashtra, India; 2 University of Ibadan, Ibadan, Nigeria

I commend the authors for the dedication to ensuring the comments and suggestions were considered and used to strengthen the manuscript.

 The authors have addressed majority of the concerns raised.

Is the review written in accessible language?

Partly

Are all factual statements correct and adequately supported by citations?

Partly

Are the conclusions drawn appropriate in the context of the current research literature?

Partly

Is the topic of the review discussed comprehensively in the context of the current literature?

Partly

Reviewer Expertise:

Infectious diseases management, vector-borne disease control, One Health, Environmental Health, Biomedical research

I confirm that I have read this submission and believe that I have an appropriate level of expertise to confirm that it is of an acceptable scientific standard.

# Reviewer report: Reviewer response for version 1

**DOI:** 10.5256/f1000research.184007.r456137

**Published:** 2026-03-05

**Authors:** Md Jisan Ahmed

**Affiliations:** 1 Sher-e-Bangla Agricultural University (SAU), Dhaka, Bangladesh

**Title: Bottom-Up Communication Approach for Effective Implementation of the One Health Initiative in Ethiopia**

**Introduction**

1. The introduction provides contextual background but does not clearly identify the specific implementation gap this paper addresses. A concise statement of the problem and the manuscript’s unique contribution is needed.
2. It is unclear whether the manuscript is a conceptual commentary, policy analysis, or evidence-based review. The authors should clarify the methodological approach used to develop the framework.
3. The argument against top-down communication is general and lacks Ethiopia-specific examples. Concrete cases or documented implementation challenges should be included.
4. The concept of bottom-up communication is introduced descriptively without reference to governance, participatory communication, or health systems frameworks. Theoretical linkage would strengthen the manuscript.
5. Ethiopian examples are presented sequentially but not analytically integrated. A brief synthesis identifying lessons or principles would improve coherence.
6. The mention of Mpox lacks epidemiological data or citations specific to Ethiopia. Clarify its relevance and public health significance.
7. The levels at which the bottom-up approach operates (community, district, national) are not explicitly defined, which limits clarity.

**The Rationale for Bottom-Up Communication in One Health**

1. The initial communication definition is generic and somewhat repetitive. Focus more on Ethiopia-specific challenges.
2. The section is persuasive but normative. Including empirical examples from Ethiopian One Health initiatives would strengthen credibility.
3. The rationale would benefit from grounding in participatory governance or health systems decentralization frameworks.
4. Statements on trust-building and improved compliance during emergencies need empirical or citation support.
5. The claim that local actors are underrepresented in formal surveillance systems requires more elaboration, including structural barriers and examples.

**Institutional Considerations and Policy Alignment**

1. Weak linkages and fragmentation are described, but the reasons these persist (political, administrative, fiscal) are not explored.
2. While HEP is presented as a practical entry point, limitations such as workload, training gaps, and coordination challenges should be acknowledged.
3. The proposal to establish teams is relevant but lacks detail on structure, reporting lines, and accountability mechanisms.
4. Fragmented data systems and siloed budgets are noted, but explanation on how joint planning mechanisms would overcome these challenges is missing.
5. Updating laws and empowering regional bureaus is suggested without discussion of feasibility, timelines, or potential resistance.

**Challenges and Mitigation Strategies**

1. Challenges are listed clearly but without prioritization or distinction between structural and operational constraints.
2. Infrastructure gaps, workforce shortages, and surveillance fragmentation need stronger empirical backing.
3. Suggested solutions such as digital expansion and legal reforms are ambitious; cost, sustainability, and phased implementation should be discussed.
4. The mitigation strategies are not explicitly tied to the earlier bottom-up communication framework.
5. It is unclear which regulatory instruments require revision and how accountability would be ensured.

**Bottom-Up Communication Framework**

1. The framework is text-heavy; consider summarizing the four core components more succinctly.
2. The diagram is repeatedly referenced, but dynamic interactions, accountability loops, and decision triggers are not clearly described.
3. Multiple examples are included, but measurable outcomes, lessons learned, or limitations are not critically discussed.
4. Resource needs, sustainability, and operational challenges for new dialogues, champions, and tools are not discussed.
5. Conceptual overlap exists between woreda and regional roles; distinct mandates should be clarified.
6. Coordination authority, reporting structures, and performance monitoring mechanisms are not clearly defined.

**Hierarchies for Bottom-Up Communication in the One Health Approach**

1. While the hierarchy is described, functional dynamics, feedback loops, and decision-making processes across levels are not critically analyzed.
2. Most discussion restates the flowchart; insights on bottlenecks, redundancies, or governance challenges should be included.
3. Success indicators, efficiency measures, and responsiveness benchmarks are absent.
4. Accountability of national stakeholders for implementing regional recommendations is not explicit.
5. Potential obstacles such as resource constraints, regional disparities, and technology gaps are not discussed.

**Conclusion and Recommendations**

1. While summarizing key points, the conclusion does not critically address challenges, limitations, or feasibility considerations.
2. The recommendations are comprehensive but presented as a list. Highlighting priority or phased actions would improve usability.
3. Linkage between recommendations and the four-component bottom-up framework should be made explicit.
4. Ambitious suggestions on legal reform, infrastructure, and multisectoral coordination lack discussion of timelines, responsible institutions, and constraints.
5. Mechanisms for long-term adoption, monitoring, and scaling of bottom-up practices are not sufficiently described.

**Overall Recommendation: Major Revision**

Is the review written in accessible language?

Partly

Are all factual statements correct and adequately supported by citations?

Partly

Are the conclusions drawn appropriate in the context of the current research literature?

Partly

Is the topic of the review discussed comprehensively in the context of the current literature?

Partly

Reviewer Expertise:

One Health, Infectious disease, AMR, Public Health, Zoonosis, Microbiology, Genomics

I confirm that I have read this submission and believe that I have an appropriate level of expertise to confirm that it is of an acceptable scientific standard, however I have significant reservations, as outlined above.

# Response

**Published:** 2026-03-09

**Authors:** Tadesse Shiferaw Chekol

**Affiliations:** One Health Research Division, Armauer Hansen Research Institute, Addis Ababa, Addis Ababa, Ethiopia

**Point-by-Point Response to Reviewer; For Reviewer 2**

 We sincerely thank the reviewer for the constructive and detailed comments. We have revised the manuscript substantially to clarify the conceptual approach, strengthen the analytical framing, incorporate Ethiopia-specific examples, and improve the overall clarity and scientific rigor. All revisions are indicated using Track Changes in the revised manuscript. Below, we respond to each comment.

**Section: Introduction**

**Reviewer Comment 1:** “The introduction does not clearly identify the implementation gap.”

**Response**

 We appreciate this observation. The introduction has been revised to explicitly state the implementation gap, namely the insufficient integration of community-level communication channels into Ethiopia’s One Health governance structure. The revised text clarifies that the paper proposes a bottom-up communication framework to address this gap.

**Reviewer Comment 2:** “Unclear whether the manuscript is conceptual or evidence-based.”

**Response**

 The manuscript now clearly states that it is a conceptual and policy-oriented analysis informed by a narrative review of literature and policy documents.

**Reviewer Comment 3:** “Need Ethiopia-specific examples.”

**Response**

 Examples of communication bottlenecks within Ethiopia’s surveillance and reporting structures have been added.

**Reviewer Comment 4:** “Lack of theoretical framework.”

**Response**

 The revised manuscript now references participatory governance and decentralized health system frameworks.

**Reviewer Comment 5:** “Ethiopian examples need synthesis.”

**Response**

 A synthesis paragraph has been added summarizing lessons from Ethiopian experiences.

**Reviewer Comment 6:** “Mpox reference lacks context.”

**Response**

 The Mpox statement now includes epidemiological context.

**Reviewer Comment 7:** “Operational levels unclear.”

**Response:** The operational levels of the framework have now been defined.

**Section: Rationale for Bottom-Up Communication in One Health**

**Reviewer Comment 8:** “The initial communication definition is generic and repetitive. Focus more on Ethiopia-specific challenges.”

**Response**

 Thank you for this comment. The opening paragraph of this section has been revised to reduce generic discussion and focus more specifically on communication challenges within Ethiopia’s One Health governance and surveillance systems.

**Reviewer Comment 9:** “Include empirical examples from Ethiopian One Health initiatives.”

**Response**

 Examples from Ethiopian multisectoral collaboration and community-based surveillance have been incorporated to strengthen the empirical basis of the discussion.

**Reviewer Comment 10:** “Ground the rationale in participatory governance or decentralization frameworks.”

**Response**

 Relevant theoretical frameworks on participatory governance and decentralized health systems have now been incorporated.

**Reviewer Comment 11:** “Statements on trust-building and compliance need citation support.”

**Response**

 The manuscript now includes supporting evidence from the literature on community engagement and risk communication.

**Reviewer Comment 12:** “Local actors underrepresented in surveillance systems require elaboration.”

**Response**

 The manuscript now explains structural barriers that limit the participation of local actors in formal surveillance systems.

**Section: Institutional Considerations and Policy Alignment**

**Reviewer Comment 13:** “Reasons for fragmentation are not explored.”

**Response**

 The revised manuscript now explains political, administrative, and financial factors contributing to fragmented coordination.

**Reviewer Comment 14:** “Limitations of the Health Extension Program should be acknowledged.”

**Response**

 A paragraph acknowledging operational challenges has been added.

**Reviewer Comment 15:** “Proposed teams lack structural details.”

**Response: **The manuscript now clarifies institutional structure and accountability.

**Section: Challenges and Mitigation Strategies**

**Reviewer Comment 16:** “Challenges not prioritized.”

**Response:** Challenges are now categorized into structural, operational, and resource-related constraints.

**Reviewer Comment 17:** “Infrastructure gaps and workforce shortages need stronger backing.”

**Response:** Supporting evidence and explanation have been added.

**Reviewer Comment 18:** “Solutions appear overly ambitious.”

**Response:** Phased implementation and sustainability considerations are now discussed.

**Section: Bottom-Up Communication Framework**

**Reviewer Comment 19:** “Framework too text-heavy.”

**Response:** A summary table has been added.

**Section: Hierarchies for Bottom-Up Communication**

**Reviewer Comment 20–24 together:** Hierarchy discussion needs deeper analysis.

**Response:** Additional discussion of feedback loops and governance challenges has been added.

**Reviewer Comment 25:** “Conclusion lacks limitations.”

**Response:** A limitations paragraph has been added.

**Reviewer Comment 26–30:** “Recommendations need prioritization.”

**Response:** Recommendations have been reorganized into phased actions in the revised version of the manuscript.

# Reviewer report: Reviewer response for version 1

**DOI:** 10.5256/f1000research.184007.r413067

**Published:** 2025-09-29

**Authors:** Chinenyenwa M. D. Ohia

**Affiliations:** 1 Biosciences, Indian Institute of Technology Bombay (Ringgold ID: 29491), Mumbai, Maharashtra, India; 2 University of Ibadan, Ibadan, Nigeria

1. Short summary of the article

 The paper presents a *bottom-up communication framework* intended to improve implementation of Ethiopia’s One Health initiative. The manuscript outlines the rationale for privileging community-level voices (community dialogues, participatory surveillance, capacity building of frontline actors) and suggests institutional measures (integration with Health Extension Program, woreda-level interdisciplinarity, legal/regulatory alignment) to enable vertical and horizontal information flows. The article references national structures (One Health Steering Committee, One Health Strategic Plan) and gives examples from Ethiopia (pilot activities, community surveillance in Somali region, OHSRS) to support the argument. The authors propose a four-part model (inclusive community engagement, participatory surveillance, localized capacity building, and dynamic feedback mechanisms) and discuss practical challenges (infrastructure, HR gaps, data fragmentation) and mitigation strategies.

 2. Strengths

- Topic is highly relevant for Ethiopia and other low- and middle-income countries with close human–animal–environment interdependence.
- The paper links communication strategy explicitly with operational structures (HEP, woreda offices) and national policy instruments, which is important for implementation.
- The framework is pragmatic and focuses on local ownership, community dialogues, and participatory surveillance — all consistent with global One Health thinking.

3. Expanding on my answers to the questions above.

 a. Is the topic discussed comprehensively in the context of the current literature? — Partly.

 b. Are all factual statements correct and adequately supported by citations? — Partly. (Many factual claims have citations, but some assertions lack up-to-date or specific references or omit key supporting evidence.)

 c. Is the review written in accessible language? — Partly **.** (Overall readable and organized, but there are awkward sentences and places that need clearer phrasing and tighter editing.)

 d. Are the conclusions drawn appropriate in the context of the current research literature? — Partly. (Conclusions are plausible but at times overly confident given the article’s primarily conceptual / descriptive nature and lack of empirical validation.)

 For the scientific soundness of the paper: I highlight below some important points the authors must provide in the article.

 a. The manuscript cites relevant documents and examples but gives no clear explanation of how the literature was selected.

 b. Factual statements, evidence & referencing: Several factual claims are referenced but others lack precise citations. Examples: statements about the performance of specific pilots (e.g., “Jigjiga University’s pilot activities”) and statements about the success of integrating priority zoonotic surveillance into polio programs need concrete citations or links to evaluation reports. Also, some assertions are temporally sensitive (e.g., “Mpox is another newly emerging health threat”) and need dates/references.

 c. Equity, gender, and sociocultural factors: The paper mentions pastoralist contexts but does not sufficiently address gender dynamics, minority and marginalized groups, or how bottom-up approaches must be adapted for literacy, languages, and power asymmetries. The authors can add a paragraph on equity and inclusion: how to ensure OHCDs include women, youth, marginalized pastoral communities, and how to manage local power imbalances (e.g., facilitators training, quota for representation).

 d. Language, structure, and editing: Although readable there are awkward sentences and occasional grammatical errors. Long paragraphs could be shortened to improve clarity. And careful editing and avoiding unnecessary repetitions

 e. Limitations and balanced tone: The manuscript occasionally reads confidently as if the framework has been proven effective. Given it is conceptual and largely descriptive, the tone should be more cautious about expected outcomes. Consider adding a limitation section discussing limitations of the topic.

 f. The authors may consider adding a table or infographic summarizing the framework, entry points, and suggested indicators to aid visual uptake by readers.

 g. The authors should address the below to make the article scientifically sound

1. Provide up-to-date and specific references for factual claims (especially program/pilot claims).
2. State limitations clearly and tone down claims of proven effectiveness—frame the paper as a framework/perspective requiring empirical validation.
3. Improve referencing and include recent documents (e.g., national One Health strategic documents, EPHI risk communication strategy, One Health Joint Plan of Action).

Is the review written in accessible language?

Partly

Are all factual statements correct and adequately supported by citations?

Partly

Are the conclusions drawn appropriate in the context of the current research literature?

Partly

Is the topic of the review discussed comprehensively in the context of the current literature?

Partly

Reviewer Expertise:

Infectious diseases management, vector-borne disease control, One Health, Environmental Health, Biomedical research

I confirm that I have read this submission and believe that I have an appropriate level of expertise to confirm that it is of an acceptable scientific standard, however I have significant reservations, as outlined above.

# Response

**Published:** 2026-03-09

**Authors:** Tadesse Shiferaw Chekol

**Affiliations:** One Health Research Division, Armauer Hansen Research Institute, Addis Ababa, Addis Ababa, Ethiopia

**Point-by-Point Response to Reviewer; For Reviewer 1**

**Reviewer Comment 1:** “The manuscript cites relevant documents and examples but gives no clear explanation of how the literature was selected.”

**Response:**

 We thank the reviewer for highlighting the need for greater transparency regarding literature selection. We have now added a short subsection in the Introduction describing the narrative review approach used to identify relevant literature, including searches of international One Health frameworks, Ethiopian national policy documents, and peer-reviewed literature.

**Reviewer Comment 2:** “Several factual claims lack precise citations, particularly statements about pilot activities (e.g., Jigjiga University pilot) and integration of zoonotic surveillance into polio programs.”

**Response:**

 We appreciate this important observation. We have reviewed the manuscript and added additional references to support statements related to pilot activities, participatory surveillance initiatives, and integration of zoonotic disease surveillance into existing health programs. We also updated temporally sensitive statements, such as references to emerging diseases by adding dates and appropriate citations.

**Reviewer Comment 3:** “Equity, gender, and sociocultural factors are insufficiently addressed.”

**Response:**

 We agree that equity and inclusion are essential components of effective bottom-up communication. We have added a new paragraph discussing gender dynamics, participation of marginalized pastoral communities, linguistic diversity, literacy barriers, and mechanisms to ensure inclusive representation in One Health Community Dialogues (OHCDs).

**Reviewer Comment 4:** “Language and structure require improvement; avoid repetition and shorten long paragraphs.”

**Response:**

 We carefully edited the manuscript for language clarity and readability. Several long paragraphs were shortened, redundant statements were removed, and grammar was revised throughout the manuscript.

**Reviewer Comment 5:** “The manuscript sometimes reads as if the framework has been proven effective. The tone should be more cautious.”

**Response:**

 We agree with this suggestion. The manuscript has been revised to clarify that the proposed framework is conceptual and policy-oriented rather than empirically validated. Statements implying proven effectiveness were modified to emphasize the framework’s potential utility and need for empirical evaluation.

**Reviewer Comment 6:** “Add a limitation section discussing limitations of the topic.”

**Response:**

 A new Limitations subsection has been added to acknowledge that the framework is conceptual and that empirical validation, operational testing, and evaluation of communication outcomes are required.

**Reviewer Comment 7:** “Consider adding a table or infographic summarizing the framework.”

**Response:**

 We appreciate this helpful suggestion. A new table summarizing the proposed bottom-up communication framework, including its components, operational entry points, and potential monitoring indicators, has been added to enhance clarity and practical application.
